# Supplementary material for: Effects of FODMAPs and Gluten on Gut Microbiota and Their Association with the Metabolome in Irritable Bowel Syndrome: A Double-Blind, Randomized, Cross-Over Intervention Study
Source: Nutrients. 2023 Jul 5;15(13):3045. doi: 10.3390/nu15133045 (PMC10346910; doi:10.3390/nu15133045)
Supplement: Supplementary file 1 [file nutrients-15-03045-s001.zip › nutrients-2419834-supplementary.pdf]

FODMAPs but not gluten alter the gut microbiota and associate with the metabolome in irritable bowel syndrome

Nordin et al.

Online Supplementary Material

Supplementary material

Supplementary Table S1. Daily intake of rice porridge (3 servings per day) with FODMAPs, gluten, and placebo.

		Daily rice porridge intake		
	**Cake (g)**	**FODMAPs (g)**	**Gluten (g)**	**Placebo (g)**
Fructose	19.5	19.5	0	0
Lactose	15.7	15.7	0	0
FOS	7.0	7.0	0	0
GOS	1.5	1.5	0	0
Sorbitol	4.5	4.5	0	0
Mannitol	1.8	1.8	0	0
Gluten	17.3	0	17.3	0
Cocoa	4.0	0	0	0
Sucrose	0	0	0	18.0
Icing sugar	0	0	24.0	0
Rice flakes	0	78.0	78.0	78.0

Abbreviations: FODMAPs, fermentable oligo-, di-, monosaccharides and polyols; FOS, fructo-oligosaccharides; GOS, galacto-oligosaccharides.

Fructose (Minimum 99.5%, Engelhardt, Sweden, Caldic, Sweden)
Lactose (Minimum 99%, Engelhardt, Sweden, Caldic, Sweden)

FOS (97 ± 2%, Caldic, Sweden)

GOS (69%, plus 23% lactose, 5% glucose and galactose, FrieslandCampina Ingredients, Netherlands)

Sorbitol (minimum 97%), mannitol (minimum 98%, Roquette, France)

Gluten (78%, Lantmännen, Sweden)

Sucrose and icing sugar (Engelhardt, Sweden)

Cocoa (Fazer, Finland)

Rice flakes (Quaker, Orkla Foods Sverige AB, Sweden)

Supplementary Table S2. Nutritional contents of rice porridges (FODMAPs, gluten, and placebo).

	Cake		Rice porridge with (per 100 g)			Daily intake of rice porridge (3 servings) with		
	per 100 g	per serving	FODMAPs	Gluten	Placebo	FODMAPs	Gluten	Placebo
Energy (kcal)	349.1	275.4	397.6	401.2	397.6	492.7	472.9	372.7
Protein (g)	22.9	18.1	4.7	18.1	5.9	5.8	21.3	5.5
Ash (g)	0.6	0.5	0.3	0.4	0.4	0.4	0.5	0.4
Fat (g)	2.5	2.0	0.5	1.7	0.7	0.6	1.9	0.7
TC (g)	58.7	46.3	93.7	78.5	91.8	116.1	92.5	86.1
Fructose (g)	24.5	19.3	17.0	< 0.04	< 0.04	21.1	< 0.04	< 0.04
Lactose (g)	18.3	14.4	12.2	< 0.04	< 0.04	15.1	< 0.04	< 0.04
FOS (g)	8.7	6.9	4.7	0.4	0.3	5.8	0.5	0.2
GOS (g)	2.4	1.9	1.5	< 0.03	< 0.03	1.9	< 0.03	< 0.03
Sorbitol (g)	5.2	4.1	3.3	< 0.04	< 0.04	4.1	< 0.04	< 0.04
Mannitol (g)	2.1	1.7	1.4	< 0.04	< 0.04	1.7	< 0.04	< 0.04
DF (g)	1.6	1.2	0.9	1.3	1.1	1.1	1.6	1.0

Abbreviations: DF, dietary fiber; FODMAPs, fermentable oligo-, di-, monosaccharides and polyols; FOS, fructo-oligosaccharides; GOS, galacto-oligosaccharides; TC, total carbohydrates.

	Analysis	Method
Energy	Calculated by authors	1 g carbohydrates = 4 calories, 1 gram protein = 4 calories, 1 g fat = 9 calories
Protein	Eurofin	Nitrogen, determination in foods based on Kjeldahl (Nordic Committee on Food Analysis (NMKL) 6, 4th ed., 2003)
Ash	Eurofin	Ash, gravimetric determination in foods (NMKL 173, 2nd ed., 2005)
Fat	Eurofin	Fat, determination in foods. (NMKL 160, 1998)
TC	Calculated by authors	By difference: 10 (weight in grams) - [protein + fat + water + ash]
Fructose	Eurofin	the Association of Official Agricultural Chemists (AOAC) 982.14, mod.
Lactose	Eurofin	AOAC 982.14, mod.
FOS	Swedish University of Agricultural Sciences, Uppsala, Sweden	AOAC method 999.03
GOS	Eurofin	AOAC 2001.02
Sorbitol	Eurofin	High-performance liquid chromatography
Mannitol	Eurofin	High-performance liquid chromatography
DF	Swedish University of Agricultural Sciences, Uppsala, Sweden	AOAC method 994.13, with modifications by Andersson et al. (1999)

Supplementary Table S3. Genera and metabolites selected from Random Forest analysis reflecting the systematic difference in molecular profiles between the FODMAP and placebo interventions (n=99). Further information about the metabolites is found in a previous publication*.

**Feature**	**mz_rt**
Anaerostipes	
	RP200.071749786501_129.674144197569
Unknown phenyl sulfate 2	RN242.012496521543_142.688944775961
3-Indolepropionate	RN188.071688028145_267.375085019106
Bifidobacterium	
Unknown sulfone	RP172.040366625418_55.5443380504571
Unknown phenyl sulfate 1	RN230.012617525409_184.196815071812
	RN231.033027186211_273.585695459787

Abbreviations: FODMAPs, fermentable oligosaccharides, disaccharides, monosaccharides, and polyols; RP, analyzed in positive mode; RN, analyzed in negative mode, mass to charge (mz) and retention time (rt) value are presented separated by '_'

* Nordin E; Hellström PM; Vuong E; Ribbenstedt A; Brunius C; Landberg R IBS Randomized Crossover Challenge Study: FODMAPs Alter Bile Acids, Tryptophan and Phenolic-Derived Metabolites, While Gluten Modifies Lipid Metabolism. Submitted 2023.

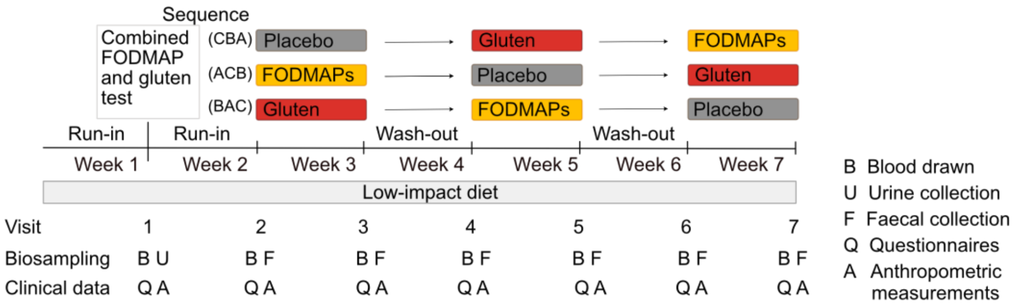

Supplementary Figure S1. Study design of the three-way double-blind, placebo-controlled randomized crossover study. Subjects were in blocks of 12 randomized into the sequences CBA, ACB, and BAC. The figure is adapted from Nordin et al*.

*Nordin E, Brunius C, Landberg R, Hellström PM. Fermentable oligo-, di-, monosaccharides, and polyols (FODMAPs), but not gluten, elicit modest symptoms of irritable bowel syndrome: a double-blind, placebo-controlled, randomized three-way crossover trial. Am J Clin Nutr 2022;115:344–52. https://doi.org/10.1093/AJCN/NQAB337.

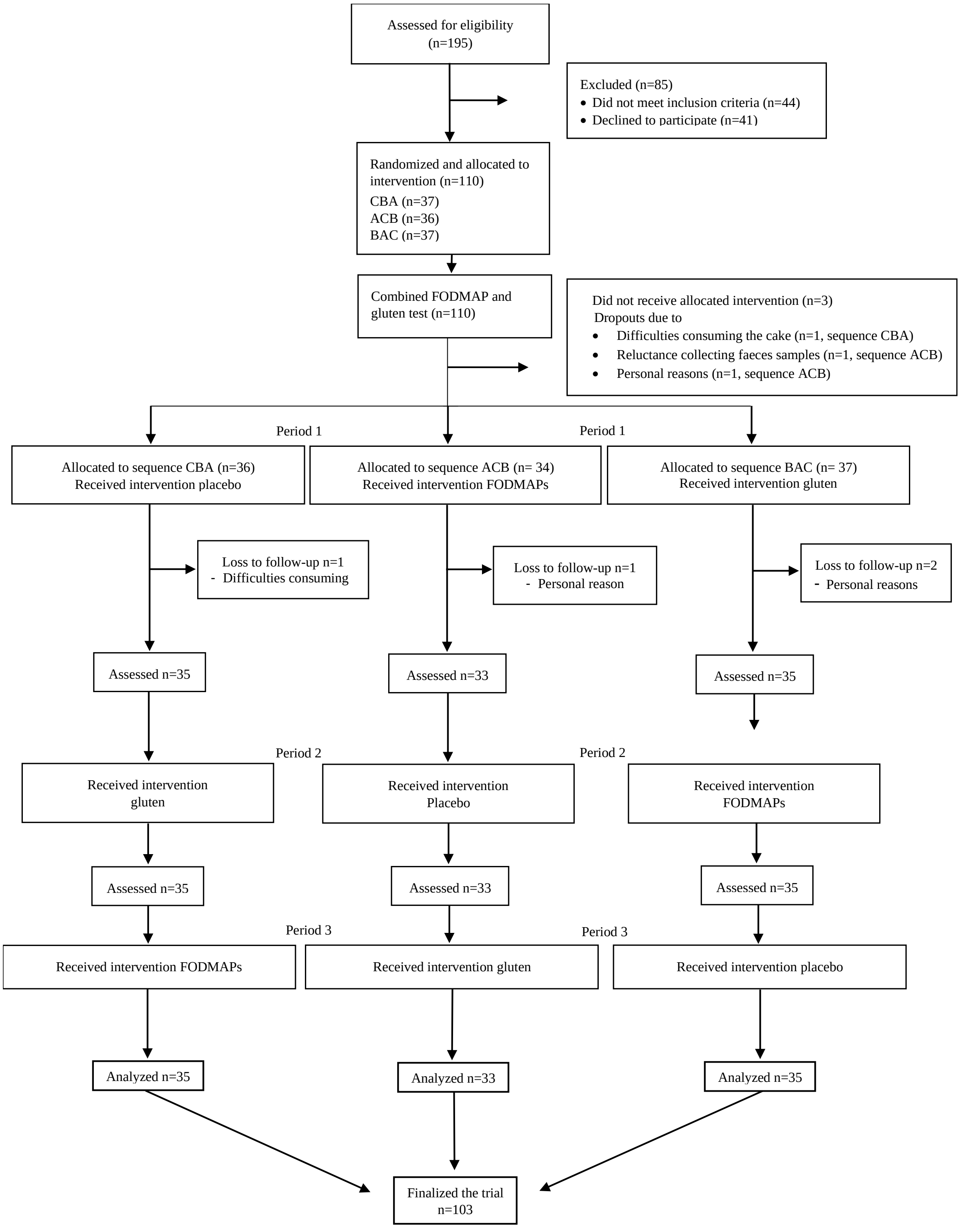

Supplementary Figure S2. Flowchart of participants in the double-blind, three-way randomized crossover trial with FODMAPs, gluten, and placebo.

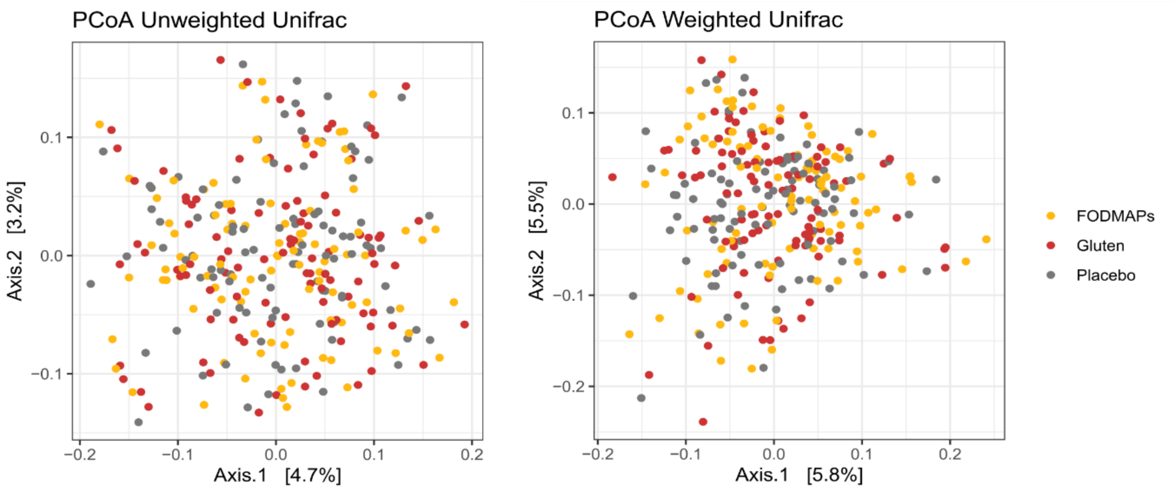

Supplementary Figure S3. Principal coordinate analysis (PCoA) [Weighted and Unweighted UniFrac] of the gut microbiota data for the interventions FODMAPs, gluten and placebo (n=100).

Abbreviation: FODMAPs, fermentable oligosaccharides, disaccharides, monosaccharides, and polyols

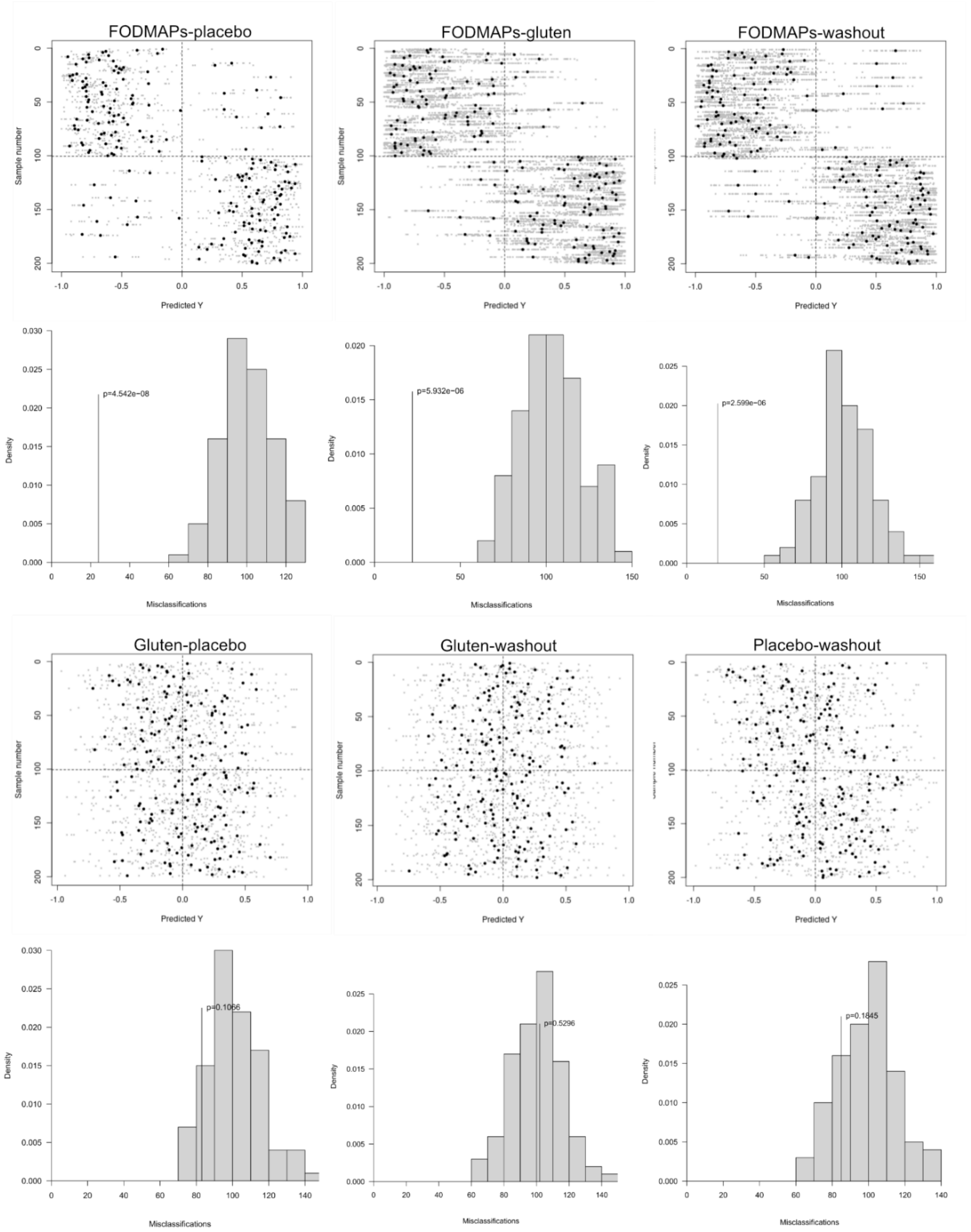

Supplementary Figure S4. Due to dependency in the repeated measure study design, Random Forest was modelled as multilevel analysis. A clear separation is seen for the FODMAP-related models in the top row, indicating systematic differences in the gut microbiota, meanwhile for the models for gluten and placebo in the third row, no separation was found. To assure results were not due to overfitting, permutation tests were performed (rows 2 and 4), assuring robust results.

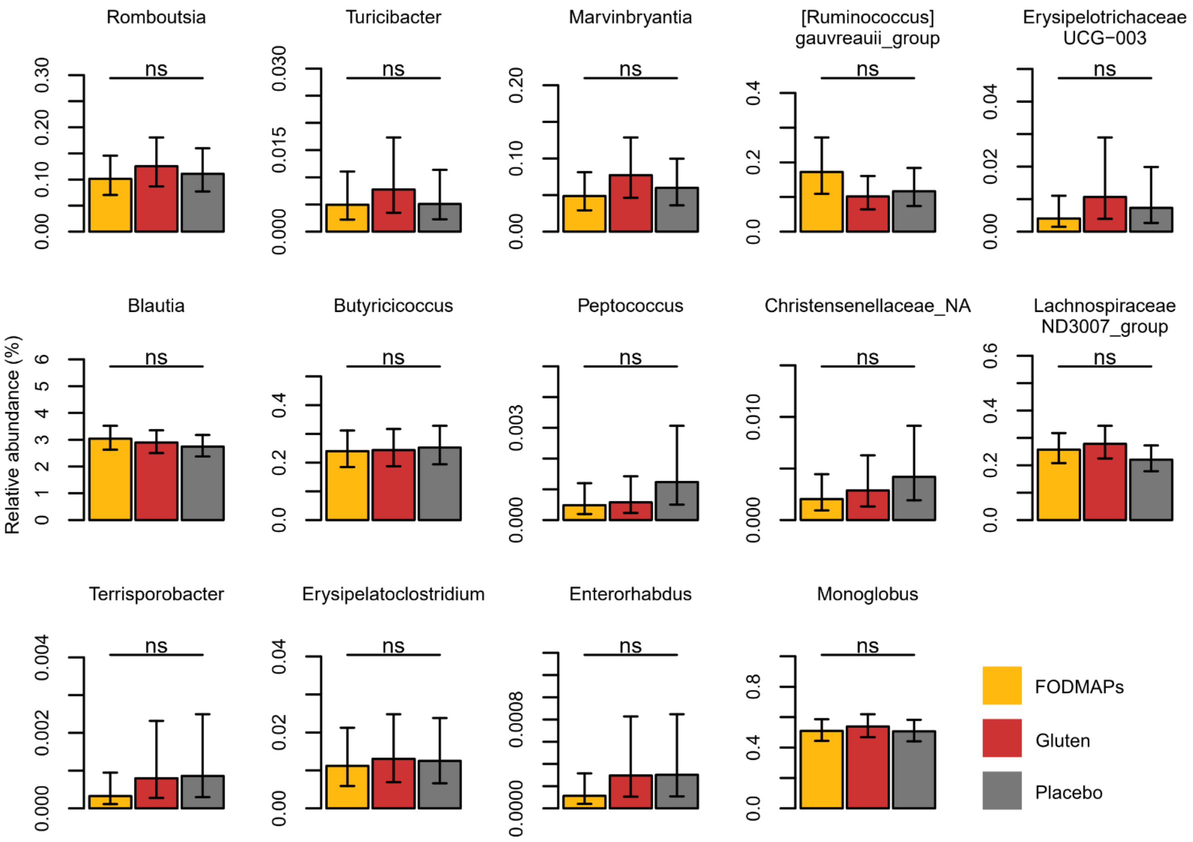

Supplementary Figure S5. Bacterial genera selected from Random Forest modelling for FODMAP-related models (FODMAPs vs placebo, FODMAPs vs washout and FODMAPs vs Gluten) with non-significant difference in relative abundance (%) between the interventions (FODMAPs, gluten and placebo) in univariate analysis (n=100). Data presented as estimated marginal means and 95 % confidence interval.

Abbreviation: FODMAPs, fermentable oligosaccharides, disaccharides, monosaccharides, and polyols

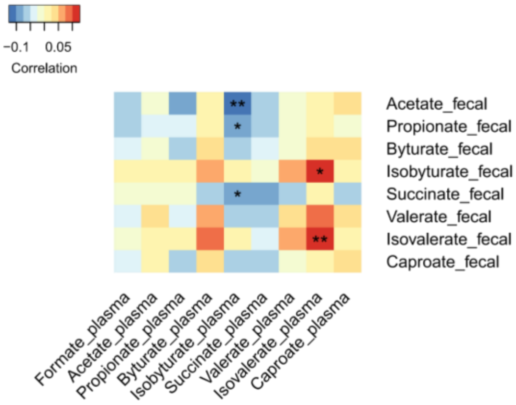

Supplementary Figure S6. Partial Spearman correlation for plasma and fecal short-chain fatty acids, all available datapoints included, adjusted for age, sex and identity (n=91, datapoint=482).
* = $p<0.05$, ** = $p<0.01$

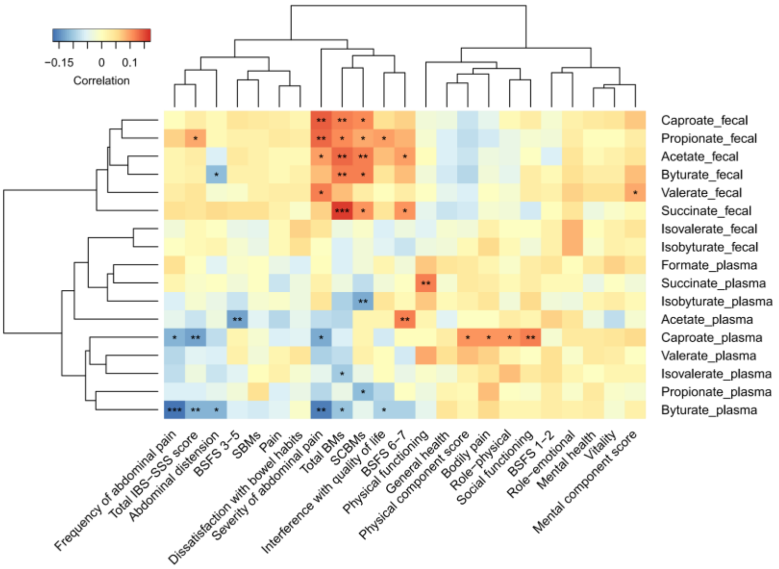

Supplementary Figure 7. Partial Spearman correlation between fecal and plasma short-chain fatty acids and the questionnaires IBS-SSS, Short Form 36 version 2 (health and quality of life), and the bowel diary, all available datapoints included, adjusted for age, sex, and identity (n=91, datapoints=482).

* = p<0.05, ** = p<0.01, *** = p<0.001

Abbreviations: BM, Bowel movements; BSFS, Bristol Stool Form Scale; Irritable Bowel Syndrome- Severity Scoring System; SBMs, spontaneous bowel movement; SCBMs, spontaneous complete (a sensation of complete evacuation) bowel movement; Pain, abdominal pain linked with bowel emptying

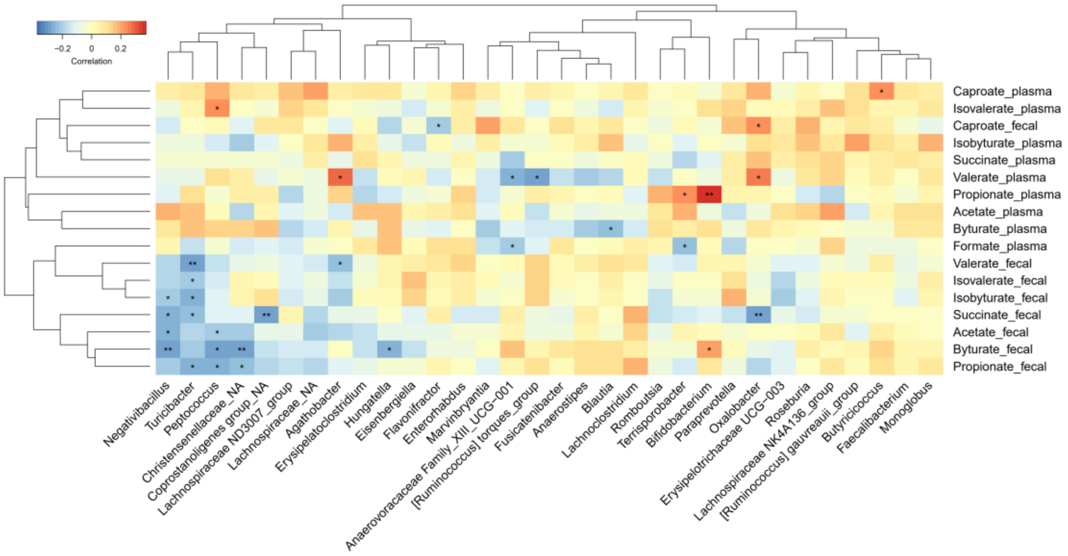

Supplementary Figure 8. Partial Spearman correlation between bacterial genera selected from Random Forest modelling for FODMAP (fermentable oligosaccharides, disaccharides, monosaccharides, and polyols)-related models (FODMAPs vs placebo, FODMAPs vs washout, and FODMAPs vs gluten) and fecal and plasma short-chain fatty acids, adjusted for age and sex (n=82).

* = p<0.05, ** = p<0.01

Abbreviation: FODMAPs, fermentable oligosaccharides, disaccharides, monosaccharides, and polyols

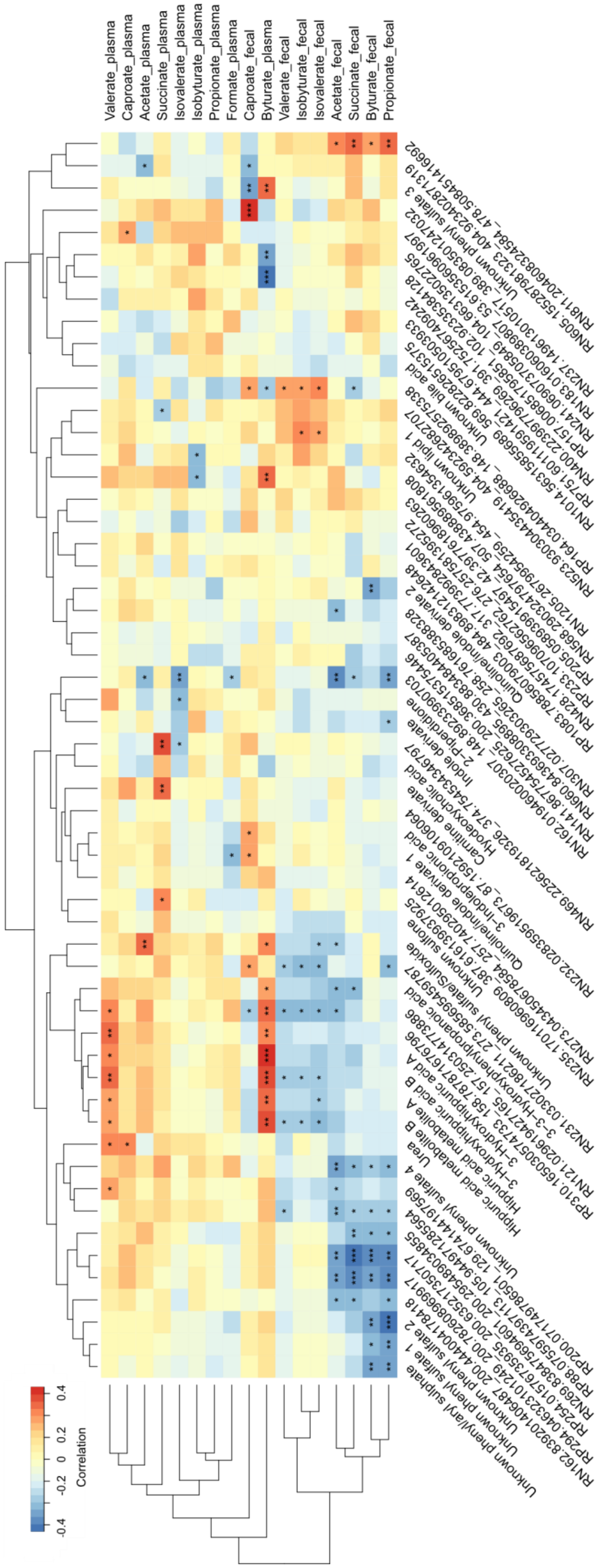

Supplementary Figure S9. Partial Spearman correlation for metabolites selected from Random Forest modelling for the FODMAP vs placebo model and fecal and plasma short-chain fatty acids, adjusted for age and sex (n=82). * = p<0.05, ** = p<0.01, *** = p<0.001

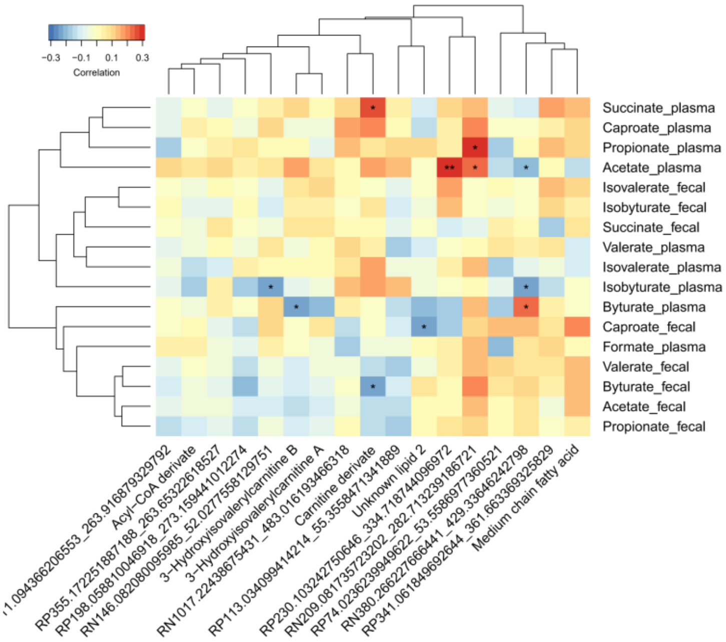

Supplementary Figure S10. Partial Spearman correlation for metabolites selected from Random Forest modelling for the gluten versus placebo model and fecal and plasma short-chain fatty acids, adjusted for age and sex (n=85).

* = p<0.05, ** = p<0.01
